# Gestational hypertensive disorders and retinal microvasculature: the Generation R Study

**DOI:** 10.1186/s12916-017-0917-2

**Published:** 2017-08-14

**Authors:** Laura Benschop, Sarah Schalekamp–Timmermans, Jeanine E. Roeters van Lennep, Vincent W. V. Jaddoe, Tien Yin Wong, Carol Y. Cheung, Eric A. P. Steegers, Kamran M. Ikram

**Affiliations:** 1 Department of Obstetrics and Gynecology, Erasmus Medical Center, Wytemaweg 80, PO Box 2040, 3000 CA Rotterdam, the Netherlands; 2 Department of General Medicine, Erasmus Medical Center, Rotterdam, the Netherlands; 3 Department of Epidemiology, Erasmus Medical Center, Rotterdam, the Netherlands; 4 Department of Pediatrics, Erasmus Medical Center, Rotterdam, the Netherlands; 5 Department of Neurology, Erasmus Medical Center, Rotterdam, the Netherlands; 6 Singapore Eye Research Institute, Singapore National Eye Centre, Singapore, Republic of Singapore; 7 Department of Ophthalmology and Visual Sciences, The Chinese University of Hong Kong, Shatin, Hong Kong

**Keywords:** Pre-eclampsia, Pregnancy induced hypertension, Microvessels, Post-pregnancy retinal imaging

## Abstract

**Background:**

Changes in the microvasculature associated with pre-eclampsia and gestational hypertension have been proposed as a potential pathway in the development of cardiovascular disease. We examined whether gestational hypertensive disorders, such as pre-eclampsia and gestational hypertension, are related to the maternal retinal microvasculature status after pregnancy.

**Methods:**

This study is part of an ongoing population-based prospective cohort study. During pregnancy and 6.2 years after the index pregnancy (90% range 5.7–7.4 years), we examined 3391 women with available information on pre-eclampsia, gestational hypertension, and retinal vascular calibers. Retinal arteriolar and venular calibers were measured in the left eye from digitized retinal photographs.

**Results:**

Women with pre-eclampsia had smaller retinal arteriolar calibers 6 years after pregnancy than women with a normotensive pregnancy (adjusted difference: –0.40 standard deviation score [SDS]; 95% confidence interval [CI]: –0.62, –0.19). For women with previous gestational hypertension, similar trends were observed (–0.20 SDS; 95% CI: –0.34, –0.05). With respect to retinal venular calibers, we did not observe consistent trends for women with previous pre-eclampsia. However, in women with previous gestational hypertension, we observed larger venular calibers (0.22 SDS; 95% CI: 0.07–0.36) than in women with a previous normotensive pregnancy. The association of gestational hypertensive disorders with retinal vessel calibers was mediated through mean arterial pressure at the time of retinal imaging.

**Conclusions:**

Compared to women with a previous normotensive pregnancy, women with pre-eclampsia and gestational hypertension show an altered status of the microvasculature 6 years after the index pregnancy. This is reflected by smaller retinal arteriolar calibers and wider retinal venular calibers. These microvascular changes may possibly contribute to the development of cardiovascular disease in later life.

**Electronic supplementary material:**

The online version of this article (doi:10.1186/s12916-017-0917-2) contains supplementary material, which is available to authorized users.

## Background

Gestational hypertensive disorders (GHD) (e.g., pre-eclampsia [PE] and gestational hypertension [GH]) affect 7% of pregnancies today [1, 2]. Both disorders are characterized by new onset of hypertension after 20 weeks of gestation and, in the case of PE, proteinuria. All features of GHD were previously believed to resolve after delivery. However, results from a large meta-analysis show that women with a history of GHD have a twofold to sevenfold increased risk of developing cardiovascular disease (CVD) in later life [3]. The exact pathophysiologic mechanism underlying this increased CVD risk remains unclear. Nevertheless, it has been shown that microvascular endothelial dysfunction as reflected by a reduced brachial artery flow-mediated dilatation persists in former pre-eclamptic women for many years after their index pregnancy [4]. These data suggest that the microvasculature may have been affected by GHD and therefore forms an important link between GHD and the development of CVD in later life.

In recent years, retinal vascular imaging has emerged as a non-invasive technique to visualize the microvasculature [5–7]. Using this technique in individuals over 50 years of age, several studies have shown that microvascular pathology is an independent contributor to the development of CVD in later life, especially in women [8]. Furthermore, a recent study examining the vasodilatory response of retinal vessels to flicker light showed that women with PE had reduced arteriolar vasodilatation during and after pregnancy compared to women with a normotensive pregnancy, indicating that microvascular dysfunction may already start early in life [9]. Examining the retinal microvasculature may provide further insight into why women with previous GHD have an increased risk for developing CVD later in life.

In the current study we investigated the association between a history of GHD and the status of the microvasculature 6 years after the index pregnancy, as reflected by retinal vascular calibers, in women between the ages of 24 to 36 years.

## Methods

### Design and study population

**Fig. 1:**
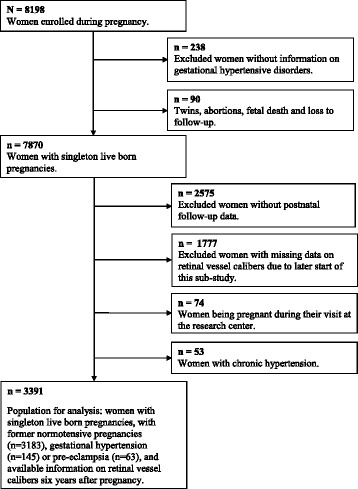
Flowchart of women involved in the study

This study was embedded in the Generation R Study, a population-based prospective cohort study from early pregnancy onwards in Rotterdam, the Netherlands [10, 11]. The study has been approved by the Medical Ethics Committee of the Erasmus University Medical Center (Erasmus MC), Rotterdam, the Netherlands, and the procedures followed were in accordance with institutional guidelines [12]. Written informed consent was obtained from all participants. For the present study we included women with a live born singleton with available information on GHD and on postnatal follow-up data. Women were excluded from the main analyses when they had a history of chronic hypertension prior to enrollment in the Generation R Study. We also excluded women who were pregnant during follow-up and women without information on retinal vascular calibers during the follow-up visit 6 years after the index pregnancy. The final population for analysis comprised 3391 women (Fig. 1).

### Gestational hypertensive disorders

The presence of doctor-diagnosed PE or GH was retrieved from hospital charts and was determined on the basis of the former criteria described by the International Society for the Study of Hypertension in Pregnancy of 2001 [13, 14]. GH was defined by a systolic blood pressure ≥140 mmHg or a diastolic blood pressure ≥90 mmHg after 20 weeks of gestation in previously normotensive women. PE was defined as de novo GH with concurrent new onset proteinuria in a random urine sample with no evidence of urinary tract infection [13].

### Maternal blood pressure and anthropometrics

Blood pressure was measured at study enrollment (median 13.2 weeks of gestation [90% range 10.6–17.0 weeks]) and 6 years after the index pregnancy (90% range, 5.7–7.4 years) as the average of two systolic and diastolic blood pressure readings, with the validated Omron 907 automated digital oscillometric sphygmomanometer (OMRON Healthcare Europe B.V., Hoofddorp, the Netherlands) [15].

All participants were in a standardized supine position to prevent differences due to position. A cuff was placed around the right upper arm. In case of an upper arm exceeding 33 cm, a larger cuff (32–42 cm) was used. The mean value of two blood pressure readings over a 5-min interval was documented for each participant. Blood pressure was measured by trained research assistants wearing normal clothing (i.e., no white coat). Mean arterial pressure (MAP) 6 years after pregnancy was calculated as the average systolic blood pressure plus two times the average diastolic blood pressure, divided by 3. Hypertension 6 years after the index pregnancy was defined by the average of two consecutive blood pressure readings, with systolic blood pressure ≥140 mmHg and/or diastolic blood pressure ≥90 mmHg or the use of antihypertensive medication [16]. Using an alternative cut-off, with systolic blood pressure ≥130 mmHg and/or diastolic blood pressure ≥80 mmHg, did not change the direction of our results but attenuated our results to non-significant levels.

At study enrollment maternal height (in centimeters) and weight (in kilograms) without shoes were measured, after which body mass index (BMI) (in kilograms/meter squared) was calculated. Identical measurements were obtained during follow-up 6 years after pregnancy. Pre-pregnancy BMI was established at enrollment through a questionnaire. Pre-pregnancy weight was highly correlated with the measured early pregnancy weight (Pearson’s correlation coefficient *r* 0.95 [*P* < 0.001]) [17].

### Retinal vascular caliber assessment

Retinal vascular calibers were assessed by taking digital retinal photographs 6 years after the index pregnancy. Unilateral and unfractionated photographs were taken of the left eye by a Topcon digital retinal camera (model TRC, NW300) while centered on the optic disk. The resolution of the images was set to 4096 and 3072 pixels. Additionally, a semi‐automatic computer‐imaging program was used to measure the six largest retinal arteriolar and venular calibers of the photos. These calibers were located one half to one disk diameter from the optic disk margin [18]. The averages of the six largest retinal arteriolar and retinal venular calibers were then summarized as central retinal arteriolar and central retinal venular equivalents [19]. The semi‐automatic computer‐imaging program that was used for computation of the central retinal arteriolar and central retinal venular equivalents was operated by two graders who were blinded to participants’ characteristics. Grader-specific standard deviation scores (SDSs) were used for both central retinal arteriolar and central retinal venular equivalents. Intraclass correlation coefficients between both graders were excellent for both retinal arteriolar calibers (0.77; 95% confidence interval [CI]: 0.69–0.84) and retinal venular calibers (0.87; 95% CI: 0.81–0.91). This suggests adequate reproducibility.

### Covariates

Information on maternal characteristics during pregnancy, including maternal age, self-reported pre-pregnancy weight, gravidity, parity, ethnicity, educational level, smoking, and chronic hypertension, was available from questionnaires repeatedly applied during pregnancy. Information on gestational age at birth, birth weight, and child sex was obtained from medical records [20, 21]. Six years after the index pregnancy, we obtained information, through questionnaires, on gravidity and parity at follow-up, medication intake, and smoking.

### Statistical analysis

We performed four types of analyses. First, a non-response analysis was performed by comparing subject characteristics between mothers with and mothers without available follow-up data 6 years after the index pregnancy (Additional file 1). Women with available follow-up data, but without information on retinal vascular calibers, were left out of the non-response analysis. Second, to reduce the possibility of potential bias associated with missing data, missing values in covariates were imputed using multiple imputation procedures. Five draws for each missing value were performed providing five substituted data points, which in turn created five completed datasets. Analyses were performed separately on each completed dataset and thereafter combined into one global result [22]. In the total population for analysis, 19.4% had missing information on pre-pregnancy BMI, 2.3% on ethnicity, 6.7% on education, 11.3% on smoking during pregnancy, 29.5% on gravidity at follow-up, 29.9% on smoking during follow-up, 2.3% on blood pressure during follow-up, and 0.2% on medication intake during follow-up. Third, differences in maternal characteristics during pregnancy and follow-up were compared between women with GHD and women with normotensive pregnancies using one-way analysis of variance (ANOVA) for continuous variables and chi-square tests for categorical variables. Fourth, associations between GHD, normotensive pregnancies, and retinal vascular calibers were assessed through linear regression. The linear regression model included covariates selected based on their associations with the outcome of interest based on previous studies or a change in effect estimate of >10% (maternal age at enrollment, ethnicity, educational level at enrollment, smoking during pregnancy, and pre-pregnancy BMI; lastly, when assessing retinal arteriolar caliber, we additionally adjusted for retinal venular caliber and vice versa. GHD are known to increase blood pressure, and blood pressure is known to be associated with smaller retinal arteriolar calibers [23]. To examine the mediating role of mean arterial blood pressure at the time of retinal imaging in the association of GHD with retinal vascular calibers, we analyzed the direct and indirect causal mediation effects through mediation analyses [24]. We used the full model, as was used for linear regression analysis, to adjust for confounding. Statistical analyses were performed using the Statistical Package for the Social Sciences version 21.0 for Windows (SPSS Inc., Chicago, IL, USA) and R version 3.3.2 (R foundation for Statistical Computing, Vienna, Austria [packages ‘foreign’, ‘rms’ and ‘mediation’]) [25].

## Results

Tables 1 and 2 show maternal characteristics during pregnancy and 6 years after the index pregnancy. Women with GHD had higher systolic and diastolic blood pressures at the start of pregnancy and 6 years later compared to women with a normotensive pregnancy. Additionally, women with a history of GHD had hypertension more often and were more likely to take cardiovascular or antihypertensive medication 6 years after pregnancy compared to women with a normotensive pregnancy.

**Table 1:** Subject characteristics by gestational hypertensive disorder (*n* = 3391)

	Normotensive pregnancy	GH	PE	*P* value
	(*n* = 3183)	(*n* = 145)	(*n* = 63)	
Maternal characteristics (pregnancy)				
Age at enrollment (years)	30.1 (5.1)	30.4 (5.1)	29.4 (5.5)	0.44
Gestational age at enrollment (weeks)	13.9 (10.9, 22.2)	13.4 (10.1, 22.9)	13.8 (10.3, 22.5)	0.15
Height (cm)	166.6 (7.4)	168.0 (7.3)	165.3 (6.8)	0.03
Pre-pregnancy weight (kg)	64.0 (50.0, 87.0)	70.0 (54.0, 114.4)	68.0 (53.2, 105.0)	<0.001
Pre-pregnancy body mass index (kg/m^2^)	22.7 (18.7, 31.5)	25.2 (20.0, 40.2)	24.4 (19.2, 37.2)	<0.001
Weight at enrollment (kg)	67.0 (52.8, 91.1)	75.0 (56.3, 112.0)	70.0 (52.0, 110.4)	<0.001
Systolic blood pressure at enrollment (mmHg)	114.8 (11.7)	126.0 (13.1)	120.4 (12.9)	<0.001
Diastolic blood pressure at enrollment (mmHg)	67.3 (9.0)	76.7 (11.3)	74.3 (9.9)	<0.001
Primiparous (no., %)	1894 (60.0)	110 (75.9)	51 (81.0)	<0.001
Non-European ethnicity (no., %)	1267 (40.8)	42 (29.0)	30 (47.6)	0.005
Lower educational level (no., %)	272 (9.2)	11 (7.9)	6 (10.0)	0.02
Smoking (no., %)	749 (26.6)	43 (32.1)	13 (21.3)	0.29
Pregnancy outcomes				
Gestational age at birth (weeks)	40.1 (37.1, 42.1)	40.1 (37.2, 42.1)	38.6 (30.9, 41.5)	<0.001
Birth weight (g)	3442.1 (539.8)	3432.8 (578.6)	2889.3 (877.4)	<0.001
Small for gestational age < p10 (no., %)	296 (9.3)	20 (13.8)	17 (27.0)	<0.001
Male sex (no., %)	1624 (51.0)	76 (52.4)	25 (39.7)	0.19

Values are percentages for categorical variables, means (SD) for continuous variables with a normal distribution, or medians (90% range) for continuous variables with a skewed distribution. Differences in baseline characteristics were tested using Student’s *t* test, ANOVA, Kruskal-Wallis, and chi-square tests. Presented values are not imputed

**Table 2:** Subject characteristics 6 years after index pregnancy by gestational hypertensive disorder (*n* = 3391)

	Normotensive Pregnancy	GH	PE	*P* value
	(*n* = 3183)	(*n* = 145)	(*n* = 63)	
Maternal characteristics (follow-up)				
Age (years)	36.7 (5.1)	37.1 (4.9)	36.0 (5.5)	0.38
Visit interval (years)	6.2 (5.7, 7.4)	6.1 (5.7, 7.6)	6.0 (5.7, 7.5)	0.30
Systolic blood pressure (mmHg)	118.1 (12.0)	128.4 (17.6)	126.5 (15.8)	<0.001
Diastolic blood pressure (mmHg)	70.0 (9.3)	78.2 (12.0)	78.5 (12.4)	<0.001
Mean arterial pressure (mmHg)	86.0 (9.6)	95.0 (13.2)	94.5 (12.9)	<0.001
Hypertension (no., %)	203 (6.4)	39 26.9)	15 (23.8)	<0.001
Primigravid (no., %)	225 (10.1)	19 (17.0)	9 (22.0)	0.003
Medication (no., %)				
Cholesterol-lowering medication	8 (0.3)	1 (0.7)	0 (0.0)	0.55
Glucose-lowering medication	13 (0.4)	2 (1.4)	1 (1.6)	0.11
Anti-hypertensives	36 (1.1)	10 (6.9)	4 (6.3)	<0.001
Cardiovascular medication	43 (1.4)	10 (6.9)	4 (6.3)	<0.001
Combined	54 (1.7)	11 (7.6)	5 (7.9)	<0.001
Smoking (no., %)	420 (18.9)	21 (18.9)	3 (7.3)	0.17
Retinal arteriolar caliber (μm)	145.8 (16.9)	141.9 (17.8)	137.8 (14.4)	<0.001
Retinal venular caliber (μm)	207.1 (22.5)	208.6 (22.5)	203.8 (19.0)	0.36
Retinal arteriolar venular ratio	0.71 (0.07)	0.68 (0.07)	0.68 (0.06)	<0.001
Weight (kg)	68.6 (53.6, 97.8)	77.0 (58.8, 121.1)	73.5 (53.3, 120.7)	<0.001
Body mass index (kg/m^2^)	24.5 (19.7, 34.5)	27.7 (21.3, 45.5)	27.6 (19.6, 43.9)	<0.001

Values are percentages for categorical variables, means (SD) for continuous variables with a normal distribution, or medians (90% range) for continuous variables with a skewed distribution. Differences in baseline characteristics were tested using Student’s *t* test, ANOVA, Kruskal-Wallis, and chi-square tests. Values are not imputed

Table 3 and Additional file 2: Figure S1A and S1B show that women with PE had smaller retinal arteriolar calibers 6 years after pregnancy than women with a previous normotensive pregnancy (age-adjusted difference: –0.49 SDS; 95% CI –0.74, –0.25). Additional adjustment for ethnicity, educational level, smoking, pre-pregnancy BMI, and retinal venular caliber did not alter these results. Women with GH also had smaller retinal arteriolar calibers than women with a previous normotensive pregnancy (fully adjusted difference: –0.20 SDS; 95% CI –0.34, –0.05). Women with previous PE did not show any difference in retinal venular calibers 6 years after pregnancy compared to those with a normotensive pregnancy. However, retinal venular calibers were larger in women with GH than in those with a previous normotensive pregnancy (0.22 SDS; 95% CI 0.07–0.36).

**Table 3:** Associations of gestational hypertensive disorders with retinal vascular calibers 6 years after index pregnancy (*n* = 3391)

	Normotensive pregnancy (*n* = 3183)	Gestational hypertension (*n* = 145) Beta (95% CI)	*P* value	Pre-eclampsia (*n* = 63) Beta (95% CI)	*P* value
Retinal arteriolar caliber (SDS)					
Full model	*Ref*	–0.20 (–0.34, –0.05)	0.007	–0.40 (–0.62, –0.19)	0.0002
Retinal venular caliber (SDS)					
Full model	*Ref*	0.22 (0.07–0.36)	0.003	0.08 (–0.13, 0.30)	0.45

*CI* confidence interval, *Ref* reference, *SDS* standard deviation score

Values are regression coefficients (95% CI) and are based on linear regression. Estimates are from multiple imputed data. Full model: adjusted for maternal age at enrollment, ethnicity, educational level at intake, smoking at intake, and pre-pregnancy BMI. Additionally, we adjusted for retinal venular caliber when assessing the outcome retinal arteriolar caliber and vice versa

The results of mediation analyses for the mediating role of MAP at the time of retinal imaging in the association of GHD with retinal vessel calibers are presented in Table 4. There was mediation by MAP in the association of both PE and GH with retinal arteriolar and retinal venular calibers.

**Table 4:** The mediation role of mean arterial pressure at the time of retinal imaging in the association of gestational hypertensive disorders with retinal vascular calibers 6 years after index pregnancy (*n* = 3391)

	Normotensive pregnancy (*n* = 3183)	Gestational hypertension (*n* = 145) Beta (95% CI)	*P* value	Pre-eclampsia (*n* = 63) Beta (95% CI)	*P* value
Retinal arteriolar caliber (SDS)					
Total effect	*Ref*	–0.20 (–0.35, –0.05)	0.01	–0.41 (–0.62, –0.21)	<0.001
Direct effect	*Ref*	–0.01 (–0.14, 0.13)	0.94	–0.21 (–0.41, –0.02)	0.03
Mediated effect	*Ref*	–0.20 (–0.26, –0.13)	<0.001	–0.20 (–0.29, –0.11)	<0.001
Retinal venular caliber (SDS)					
Total effect	*Ref*	0.22 (0.08, 0.36)	<0.001	0.08 (–0.09, 0.27)	0.39
Direct effect	*Ref*	0.16 (0.02, 0.30)	0.02	0.03 (–0.14, 0.22)	0.76
Mediated effect	*Ref*	0.06 (0.03, 0.09)	<0.001	0.05 (0.02, 0.09)	<0.001

*CI* confidence interval, *Ref* reference, *SDS* standard deviation score, *BMI* body mass index

Values are regression coefficients (95% CI) and are based on mediation analysis. Values are adjusted for maternal age at enrollment, ethnicity, educational level at intake, smoking at intake, and pre-pregnancy BMI. Additionally, we adjusted for retinal venular caliber when assessing the outcome retinal arteriolar caliber and vice versa

Finally, we tested whether the amount of previous pregnancies affected our results. No differences were observed in retinal vascular calibers in association with gravidity or parity.

## Discussion

Our study shows that women with GHD have an altered status of the retinal microvasculature 6 years after the index pregnancy compared to women with a normotensive index pregnancy. In particular, women with PE have smaller retinal arteriolar calibers than women with previous normotensive pregnancies. Additionally, women with GH have wider retinal venular calibers than women with previous normotensive pregnancies. These associations may partly be related to concurrent blood pressure, since adjustment for MAP attenuated most relationships.

During a normotensive pregnancy, the maternal cardiovascular system undergoes imperative adaptations by increasing intravascular volume, heart rate, and cardiac output [26]. Concomitantly with these cardiovascular adaptations, there is a decrease in blood pressure accompanied by physiologic vasodilatation of the microvasculature [27]. Cross-sectional results from the Growing Up in Singapore Towards Healthy Outcomes (GUSTO) study support these adaptations, showing that each 10 mmHg increase in MAP during pregnancy was associated with a significant reduction in retinal arteriolar caliber, especially for MAP ≥90 mmHg [28]. Another study, using retinal images obtained throughout pregnancy and 6 months after pregnancy of 53 normotensive women, also demonstrated changes in the retinal microvasculature over the course of a normotensive pregnancy in conjunction with blood pressure adaptations [29]. During mid-pregnancy, when blood pressure shows a physiologic decrease, retinal arteriolar and retinal venular calibers increased significantly. Nevertheless, retinal arteriolar and venular calibers returned to normal (early pregnancy) values in late pregnancy and 6 months after pregnancy. These results imply that changes in the retinal microvasculature during pregnancy are transient and mainly the consequence of concurrent physiologic blood pressure fluctuations. However, other studies in non-pregnant populations demonstrated that not only concurrent but also past elevated blood pressures are associated with retinal arteriolar narrowing [30–32]. Therefore, retinal arteriolar narrowing may not only be considered a transient response to an increased blood pressure, but also the result of cumulative exposure to hypertension over the life course. The underlying pathophysiology might be explained by accumulating endothelial damage, due to hypertension, eventually leading to endothelial dysfunction and microvascular impairment [33, 34].

This might also explain the larger retinal venular calibers in women with previous GH in our study and not in women with previous PE. GH women had a higher weight and blood pressure at the start of pregnancy than PE women. These factors have been associated with larger retinal venular calibers, possibly through local vascular inflammation and endothelial damage [35, 36]. Though we did not reach statistical significance, retinal venular calibers also tended to be larger in women with previous PE than in women with a previous normotensive pregnancy. Nevertheless, we need to be causative in interpreting this finding due to the small sample size of women with previous PE.

Even though previous studies did not examine retinal microvascular calibers both before and after GHD pregnancies, it seems likely that damage to the endothelium as a result of the GHD is not completely reversible and therefore enhances retinal microvascular changes after pregnancy. Previous studies showed through flickering response and the laser Doppler perfusion imaging technique in women with PE that retinal microvascular function is impaired both during pregnancy and up to 25 years after [9, 37]. Microvascular dysfunction therefore does not merely seem to be a disorder of pregnancy. For this reason, future research should aim to visualize the retinal microvasculature, before and at multiple time points after GHD, in order to detect if retinal microvascular abnormalities precede the onset of GHD and whether they progress after pregnancy.

Numerous studies have shown a relationship between retinal vascular changes (e.g., retinal arteriolar narrowing and retinal venular widening) and future CVD [5, 32, 38–40]. For instance, a large meta-analysis showed an increased risk of coronary heart disease (CHD) in women with wider retinal venular or narrower retinal arteriolar calibers [41]. The Atherosclerosis Risk in Communities Study (ARIC) assessed retinal microvascular calibers among men and women aged 49–73 and showed that retinal arteriolar narrowing was associated with an increased risk of CHD, myocardial infarction, congestive heart failure, and incident hypertension [32, 38, 39]. Additionally, the Beaver Dam Eye Study demonstrated that retinal arteriolar narrowing is associated with an increased 10-year risk of hypertension [42]. Our study provides evidence to support the concept that women with GHD show more unfavorable retinal microvascular calibers 6 years after pregnancy than women with previous normotensive pregnancies. As a result, it is reasonable to speculate that women with GHD might have an increased risk for future CVD.

### Strengths and limitations

Several limitations of the present studies need to be discussed. First, we did not obtain retinal vascular imaging from 33.6% of all women who came for follow-up visits 6 years after pregnancy, because retinal vascular imaging was introduced into the Generation R Study after recruitment of study subjects had already started. As this was independent of any subject characteristics, we do not expect any additional selection bias. However, there may be some loss of power due to a smaller sample size available for our analysis and hence larger confidence intervals of the reported associations. Second, compared to non-responders (43%) study participants were on average older at study enrollment, more often primiparous and of European descent, higher educated, more often non-smokers, and more often had GH. This may have led to some degree of selection bias, as the included women were relatively healthy, and may have led to an underestimation of the association between GHD and retinal microvasculature. Third, due to unavailability of pre-pregnancy data on blood pressure (as is also the case in most other studies focusing on pregnant women and cardiovascular outcomes after pregnancy), we cannot exclude the possibility that microvascular changes and hypertension preceded the onset of GHD. However, we compensated for this by excluding women with chronic hypertension. Information on chronic hypertension before pregnancy was obtained through a questionnaire during pregnancy, which was cross-checked with information from the original medical records and the Dutch obstetric database [43]. Our results did not change significantly after we performed a sensitivity analysis excluding women with hypertension in early pregnancy (13.2 weeks of gestation, 95% CI; 11.1–17.0) or women with hypertension at the time of retinal imaging. Hypertension in early pregnancy was defined as a systolic blood pressure ≥140 mmHg and/or a diastolic blood pressure ≥90 mmHg. Hypertension at the time of retinal imaging was defined as the intake of antihypertensive medication and a systolic blood pressure ≥140 mmHg and/or a diastolic blood pressure ≥90 mmHg. Lowering the cut-off for hypertension at the time of retinal imaging (systolic blood pressure ≥130 mmHg and/or diastolic blood pressure ≥80 mmHg) did not change the direction of our results, but did attenuate our results to non-significant levels. Fourth, retinal vascular calibers were not assessed before pregnancy. Therefore, microvascular changes might be due to GHD or might have predated pregnancy. Fifth, the observational nature of this study does not allow for inference of causality and does not preclude the existence of residual confounding. Sixth, information on pregnancies and GHD occurring after the index pregnancy was incomplete. The absence of these data might have affected our results. Finally, our study also has several strengths. First, this is a prospective cohort study from early pregnancy onwards with a large sample size of 3391 participants. Second, retinal images were taken and graded following standardized protocols.

## Conclusions

Our study shows that in women with GHD the microvasculature is already affected early in life. Six years after the index pregnancy, women with GHD show smaller retinal arteriolar and wider retinal venular calibers than women with a normotensive pregnancy, suggesting that the changes in the microvasculature possibly represent the pathophysiological substrate linking GHD to CVD in later life. Future research should therefore aim to investigate associations between the microvasculature and cardiovascular risk factors before and after the onset of GHD and the long-term cardiovascular outcomes in these women.

## Additional files

Additional file 1: Maternal and fetal characteristics stratified for loss to follow-up (*n* = 5966). (DOCX 15 kb)

Additional file 2: Figure S1. The association of gestational hypertensive disorders with retinal arteriolar (A) and venular (B) calibers 6 years after index pregnancy. Values are regression coefficients (95% confidence interval) and are based on linear regression models. Estimates are from multiple imputed data. We adjusted for maternal age at enrollment, ethnicity, educational level at enrollment, smoking during pregnancy, and pre-pregnancy BMI, and lastly when assessing retinal arteriolar caliber, we additionally adjusted for retinal venular caliber and vice versa. (PPTX 171 kb)

## Abbreviations

BMI: Body mass index
CHD: Coronary heart disease
CI: Confidence interval
CVD: Cardiovascular disease
GH: Gestational hypertension
GHD: Gestational hypertensive disorders
MAP: Mean arterial pressure
PE: Pre-eclampsia
SDS: Standard deviation score
