# Correction to: Gestational hypertensive disorders and retinal microvasculature: the Generation R Study

**DOI:** 10.1186/s12916-017-0946-x

**Published:** 2017-10-06

**Authors:** Laura Benschop, Sarah Schalekamp–Timmermans, Jeanine E. Roeters van Lennep, Vincent W. V. Jaddoe, Tien Yin Wong, Carol Y. Cheung, Eric A. P. Steegers, M. Kamran Ikram

**Affiliations:** 1 Department of Obstetrics and Gynecology, Erasmus Medical Center, Wytemaweg 80, PO Box 2040, 3000 CA Rotterdam, The Netherlands; 2 Department of General Medicine, Erasmus Medical Center, Rotterdam, The Netherlands; 3 Department of Epidemiology, Erasmus Medical Center, Rotterdam, The Netherlands; 4 Department of Pediatrics, Erasmus Medical Center, Rotterdam, The Netherlands; 5 Department of Neurology, Erasmus Medical Center, Rotterdam, The Netherlands; 6 Singapore Eye Research Institute, Singapore National Eye Centre, Singapore, Republic of Singapore; 7 Department of Ophthalmology and Visual Sciences, The Chinese University of Hong Kong, Shatin, Hong Kong

## Erratum

The original article [1] contained an error whereby the senior author, M. Kamran Ikram’s name was mistakenly interchanged.

This error has now been corrected.
