# Supplementary material for: Gestational hypertensive disorders and retinal microvasculature: the Generation R Study
Source: BMC Med. 2017 Aug 14;15:153. doi: 10.1186/s12916-017-0917-2 (PMC5554975; doi:10.1186/s12916-017-0917-2)
Supplement: Supplementary file 1 — Maternal and fetal characteristics stratified for loss to follow-up (n = 5966). (DOCX 15 kb) [file 12916_2017_917_MOESM1_ESM.docx]

**Additional file**

| **Additional file 1:** Maternal and fetal characteristics stratified for loss to follow-up (n=5966) | | | | | | |  |  |
| --- | --- | --- | --- | --- | --- | --- | --- | --- |
|  | | **Included in analyses** | | | **Not in analyses** | | **P-Value** | |
|  | **(n = 3391) (n = 2575)** | | | | | | | |
| **Maternal characteristics (prenatal)** | | | | | | | | |
| Age at enrollment (years) | | 30.1 (5.1) | | | 28.2 (5.5) | <0.001 | |  |
| Gestational age at enrollment (weeks) | | 13.8 (10.8, 22.3) | | | 14.4 (10.8, 23.8) | <0.001 | |  |
| Height (cm) | | 166.7 (7.4) | | | 168.6 (7.4) | 0.09 | |  |
| Pre-pregnancy weight (kg) | | 64.0 (50.0, 90.0) | | | 63.0 (49.0, 92.0) | 0.07 | |  |
| Pre-pregnancy Body Mass Index (kg/m2) | | 22.7 (18.8, 31.9) | | | 23.3 (19.2, 30.9) | 0.52 | |  |
| Weight at enrollment (kg) | | 67.0 (53.0, 93.0) | | | 67.0 (51.0, 95.0) | 0.92 | |  |
| Systolic blood pressure at intake (mmHg) | | 115.3 (12.0) | | | 114.7 (12.3) | 0.06 | |  |
| Diastolic blood pressure at intake (mmHg) | | 67.8 (9.3) | | | 67.5 (9.8) | 0.25 | |  |
| Primiparous (%) | | 61.0 | | | 55.8 | <0.001 | |  |
| Non-European ethnicity (%) | | 40.4 | | | 53.1 | <0.001 | |  |
| Lower educational level (%) | | 9.1 | | | 16.9 | <0.001 | |  |
| Smoking (%) | | 26.8 | | | 30.5 | 0.004 | |  |
| **Birth and childhood characteristics** | |  | | |  |  | |  |
| Gestational age at birth (weeks) | | 40.1 (37.1, 42.1) | | | 40.0 (36.5, 42.1) | <0.001 | |  |
| Birth weight (g) | | | 3450.0 (2540.0, 4300.0) | 3380.0 (2450.0, 4300.0) | | <0.001 | |  |
| Small for gestational age (<p10), (%) | | | 9.8 | 11.3 | | 0.07 | |  |
| Male sex (%) | | | 50.9 | 51.7 | | 0.51 | |  |
| **Pregnancy complications** | | |  |  | |  | |  |
| Gestational hypertension (%) | | 4.4 | | | 3.1 | 0.02 | |  |
| Pre-eclampsia (%) | | 1.9 | | | 2.7 | 0.07 | |  |

Values are percentages for categorical variables, means (SD) for continuous variables with a

normal distribution, or medians (90% range) for continuous variables with a skewed

distribution. Differences in baseline characteristics were tested using Students t-test,

ANOVA, Kruskal-Wallis and chi-square tests. Presented values are not imputed.
