# Supplementary material for: Gestational hypertensive disorders and retinal microvasculature: the Generation R Study
Source: BMC Med. 2017 Aug 14;15:153. doi: 10.1186/s12916-017-0917-2 (PMC5554975; doi:10.1186/s12916-017-0917-2)
Supplement: Supplementary file 2 — The association of gestational hypertensive disorders with retinal arteriolar (A) and venular (B) calibers 6 years after index pregnancy. Values are regression coefficients (95% confidence interval) and are based on linear regression models. Estimates are from multiple imputed data. We adjusted for maternal age at enrollment, ethnicity, educational level at enrollment, smoking during pregnancy, and pre-pregnancy BMI, and lastly when assessing retinal arteriolar caliber, we additionally adjusted for retinal venular caliber and vice versa. (PPTX 171 kb) [file 12916_2017_917_MOESM2_ESM.pptx]

## Slide 1
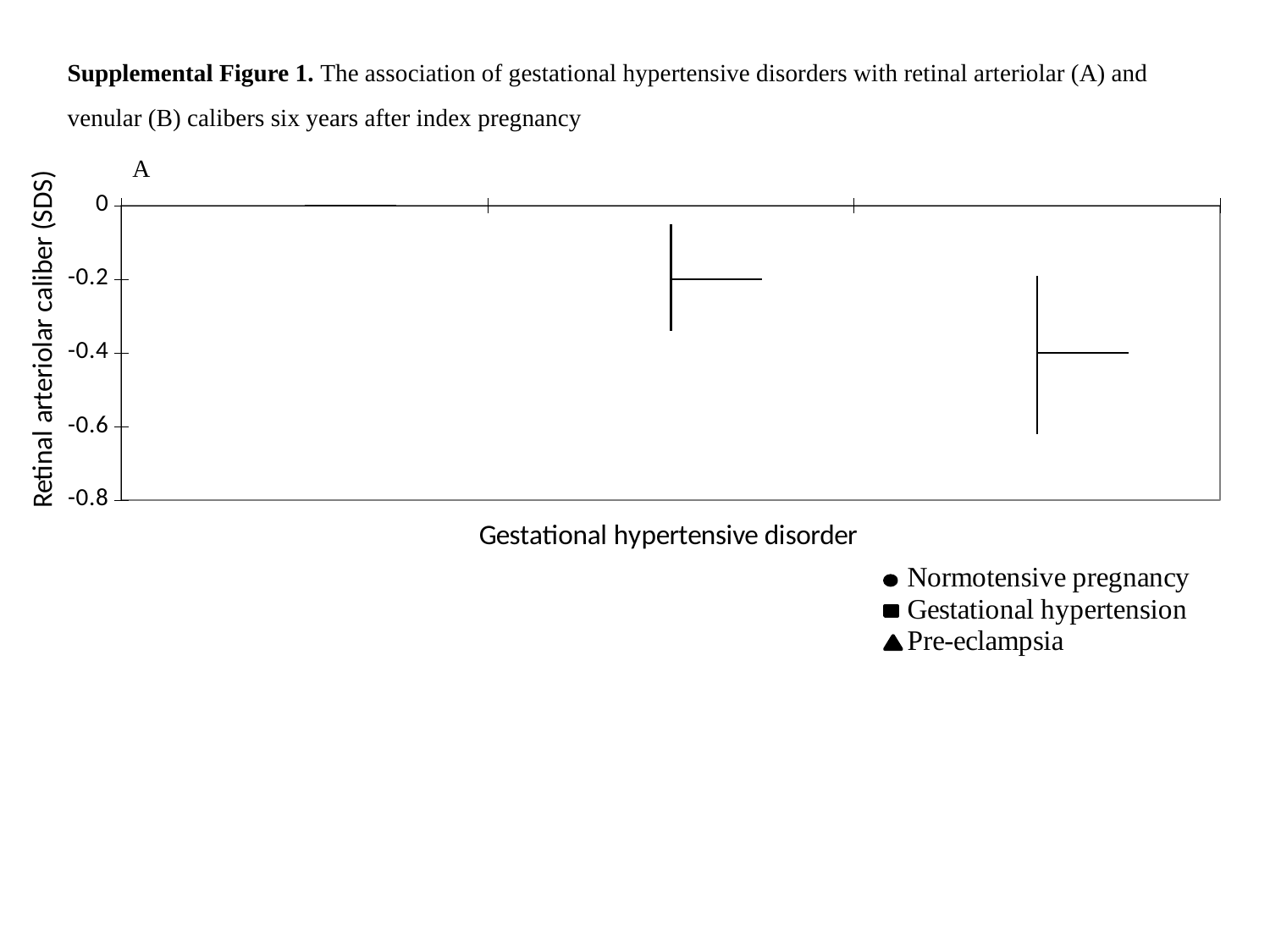

Supplemental Figure 1. The association of gestational hypertensive disorders with retinal arteriolar (A) and venular (B) calibers six years after index pregnancy
A
[unsupported chart]

## Slide 2
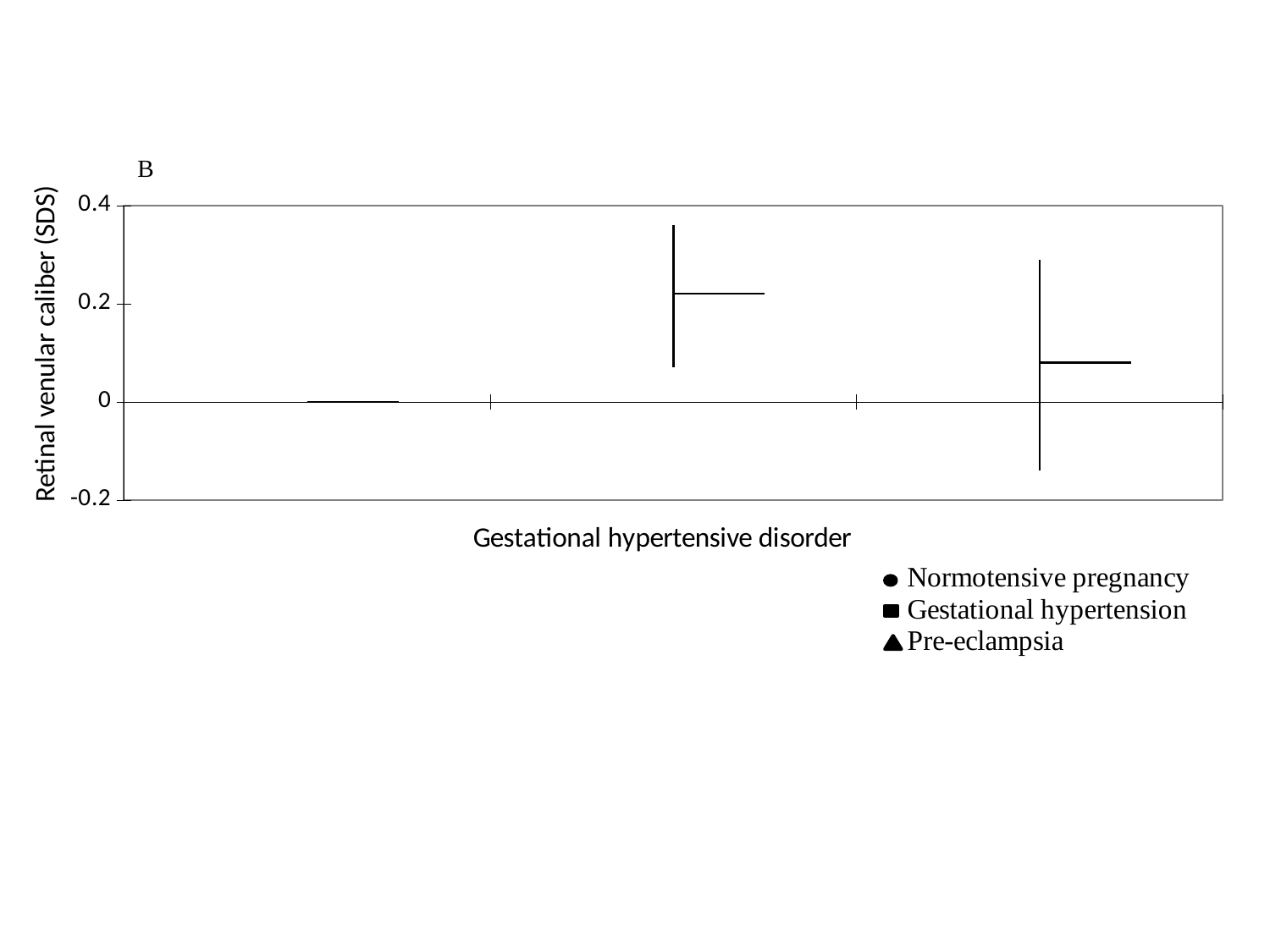

B
[unsupported chart]
